# Cross-Sectional Analysis of the Effect of Physical Activity, Nutrition, and Lifestyle Factors on Medical Students’ Academic Achievement

**DOI:** 10.7759/cureus.56343

**Published:** 2024-03-17

**Authors:** Jeffrey Neuman, Emily A Ina, Shakil O Huq, Alex Blanca, Stephanie N Petrosky

**Affiliations:** 1 Medicine, Dr. Kiran C. Patel College of Osteopathic Medicine, Nova Southeastern University, Fort Lauderdale, USA; 2 Nutrition, Dr. Kiran C. Patel College of Osteopathic Medicine, Nova Southeastern University, Fort Lauderdale, USA

**Keywords:** medical education, medical school, medical student, food and nutrition, physical activity, academic acheivement, lifestyle behavior

## Abstract

Background: Unhealthy dietary habits, decreased physical activity, poor sleep quality, and increasing levels of stress and burnout have all been identified as major concerns of medical students. Due to the rigorous environment of medical school, maintaining a well-balanced and nutritious diet is often replaced by more convenient and nutrient-poor options. Improper dietary habits and a sedentary lifestyle both play an essential role in the development of type II diabetes, obesity, hypertension, and hyperlipidemia. These unhealthy trends commonly stem from the innate drive for medical students to achieve at the highest level, sacrificing healthy lifestyle choices to maximize studying. Unfortunately, this dynamic creates a paradox where students create an unhealthy lifestyle to increase academic achievement; however, these destructive living conditions lead to a diminished sense of well-being. As a result, greater rates of burnout, comorbidities, and other maladaptive tendencies diminish success in school.

Objective: The objective of this study is to investigate the effects of lifestyle habits such as nutrition, physical activity (PA), and stress on academic performance (grade point average: GPA) among first- and second-year students at Nova Southeastern University, Kiran C. Patel College of Osteopathic Medicine (KPCOM).

Methods: A cross-sectional study was conducted on medical students (n = 161) under institutional review board (IRB) approval. The students voluntarily completed a self-reported survey containing questions about diet, exercise (frequency and type), stress, and self-reported GPA. About 800 students were invited to complete the survey between June 15, 2022, and June 28, 2022. A simple lifestyle indicator questionnaire (SLIQ) score was determined for each student based on their diet, PA, and stress responses.

Results: The results showed a significant positive correlation between SLIQ score and high academic achievement. A significant positive association was found between the low academic-achieving (LAA)(2.00-2.99 GPA) vs the high (3.60-4.00 GPA) academic-achieving (HAA) cohorts for physical activity (p* *= 0.012) as well as diet (p* *= 0.043). Further, the HAA cohort reported higher satisfaction with their mental and physical health (74% and 54%, respectively) as compared to the LAA cohort (29% each).

Conclusion: This study demonstrated positive correlations between diet and physical activity with high academic achievement. The HAA cohort recorded the highest rates of fruit/vegetable and home-cooked meal consumption and the greatest participation in light, moderate, and heavy exercise when compared to the middle academic-achieving (MAA) (3.00-3.59 GPA) and LAA cohorts. Interestingly, the HAA students also recorded the highest rates of satisfaction with their mental and physical health. As a result, there is a need to promote healthier lifestyle trends among medical students with a focus on diet and physical activity.

## Introduction

Medical students are often under the assumption that to perform better academically, they must sacrifice a healthy diet, time for physical activity, and time dedicated to maintaining their mental health. Although the demands of medical school are rigorous, current research suggests that sustaining healthy habits, such as a well-balanced diet and exercising, may have a positive impact on academic achievement and quality of life. 

Demonstrating the interplay between physical activity and nutritional status, researchers found a positive significant correlation between physically active students and consumption of water, vegetables, fruits, and carbohydrates [1]. The benefits of having a diet rich in vegetables and fruits and low consumption of high-sugar beverages go beyond the decreased likelihood of developing conditions such as type II diabetes mellitus or hypertension. Physical activity increases cerebral blood flow and functional oxygen capacity and creates a statistically significant improvement in student memory [2]. Current literature also shows that most college-aged students adopt unhealthy lifestyle habits. One study found that 37% of subjects took part in physical activity once a week, and only 7% participated daily, with lack of time (60%) and energy (26%) acting as the main deterrents [3]. Additionally, the researcher found in their study examining nutritional behavior among medical professional students that only 29% had daily fruit and vegetable consumption, while 45.6% reported snacking on potato chips and salty foods between meals. The researchers hypothesize that the minimal participation in healthy lifestyle habits is due to an overemphasis on studying and neglect of one’s physical and mental needs. Loscalzo and Giannini defined a theory behind their study as the “Study-to-Relationships Conflict,” analyzing how overstudying can impact social and school relationships [4]. Their study of 598 students found that increased time studying was positively associated with relationship impairment among their family members and friends [4]. The current researchers believe a balanced schedule can be achieved if one understands the law of diminishing marginal returns, highlighting that more time spent studying does not necessarily correlate with higher academic achievement. The idea is supported by Bin Abdulrahman et al., who found that students who can maintain a balanced schedule consisting of studying, healthy habits, and taking time for themselves are less likely to experience burnout, increase interest in learning, and optimize their academic performance [5].

There is currently extremely limited literature on US medical students and the correlation between healthy lifestyles and academic achievement, leading to a gap in understanding. The aim of this study is to determine if similar benefits of nutrition and physical activity found in current research apply to US medical students. The research design assessed whether medical students at Nova Southeastern University, Dr. Kiran C. Patel College of Osteopathic Medicine (NSU-KPCOM), who consumed a healthier diet and engaged in consistent physical activity are more likely to obtain higher academic performance. Additionally, the correlation between mental, physical, and academic satisfaction with healthy lifestyle practices was analyzed.

## Materials and methods

Study design

The design of this experiment is a cross-sectional analysis of survey data assessing nutrient-rich diets, including fruits and vegetables, physical activity frequency and type, and lifestyle satisfaction as predictive factors for the dependent variable of grade point average (GPA). The participants were osteopathic medical students (OMS-I or OMS-II) at NSU-KPCOM during the 2022-2023 academic year. A total of 161 of the possible 800 students ranging from 22 to 30 years of age participated in our survey, equaling a response rate of about 20%. We focused on OMS-I and OMS-II to provide standardization to our sample population. NSU-KPCOM curricula focus on didactic teaching in the first two years of medical school, where all students are tested and graded on the same scale. Third- and fourth-year students were excluded because they are in clinical rotations and, as a result, live variable lifestyles and are graded by different preceptors in a pass/fail manner. Thus, their academic achievement cannot be measured on the same scale. This allowed for a consistent and standardized comparison of academic achievement via the GPA. 

Data collection**

The survey consisted of four sections with a total of 18 questions. The first section of the survey asked about demographics, including academic year, gender, and age. The second section asked questions regarding nutrition consumption and frequency. including high-sugar beverages, fast food, fruits, vegetables, and home-cooked meals (at a consumption frequency of less than once per week, once per week, 2-3 times per week, 4-6 times per week, once per day, or 2 or more times per day). The third section assessed physical activity, including light exercise, moderate exercise, and vigorous exercise. Answer choices were based on the frequency of participation in each category of exercise (zero times per week, 1-3 times per week, 4-7 times per week, or eight or more times per week). The fourth section asked questions on various lifestyle habits and satisfaction including the amount of time spent studying and sleeping per week, overall stress level, satisfaction with physical and mental health, and satisfaction with current academic achievement. To indicate academic achievement, self-reported GPA was collected in the last section of the survey.

Survey data were collected via SurveyMonkey for two weeks after the initial survey distribution on June 15, 2022. A total of 161 responses were collected. After cleaning, 141 responses were included, and 20 were excluded due to lack of completion or not being enrolled as an OMS-I or OMS-II at NSU-KPCOM. Results were downloaded from SurveyMonkey for analysis in MS Excel (Microsoft Corporation, Redmond, Washington, United States). The data collected on each medical students’ health was categorized based on a modified Simple Lifestyle Indicator Questionnaire (SLIQ), which incorporates questions that solicit individuals' overall lifestyle (diet, exercise, stressors) [6]. The total tallied raw score of each category was assigned a value of 0, 1, or 2, which contributed to an overall SLIQ score that would indicate each medical student's overall health, with a higher number representing increased frequency in healthy lifestyle practices. We stratified GPA into a low academic achieving (LAA) group (2.30-2.99), a middle AA (MAA) group (3.00-3.59), and a high AA (AA) group (3.60-4.00). We used these three stratifications to correlate academic performance to the overall health of each participant based on their survey responses and overall SLIQ score. We then used the group means to conduct our analysis.

## Results

Demographics

The final analysis pool (N = 141) had a mean age of 25.62; males comprised 54 (38%) of the sample, while 84 (60%) were females, 1 (0.6%) reported as nonbinary, and 2 (1.4%) did not specify. Additionally, 117 were OMS-I (83%), and 24 (17%) were OMS-II (Table 1).

**Table 1:** Demographics and Characteristics of the Participants (N = 141) OMS: Osteopathic medical student; SD: standard deviation; n: population size

Variables	Mean ± SD or n (%)
Gender	
Males	54 (38%)
Nonbinary	1 (0.6%)
Females	84 (60%)
Unspecified	2 (1.4%)
Class	
OMS-1	117 (83%)
OMS-2	24 (17%)
Average age	25.62

Dietary factors

The effect of diet and nutrition among medical students was assessed based on questions about various food and drink consumption and frequency. An unhealthy diet was evaluated via frequent high-sugar beverage and fast-food intake. In contrast, healthier categories included intake of fruits, leafy greens, vegetables, and home-cooked meals (Table 2).

**Table 2:** The Association Between Diet and the Study Variables <1: less than once a week; 1/wk: once per week; 2-3/wk: 2-3 times per week; 4-6/wk: 4-6 times per week; 2+ / day: 2 or more times per day; GPA: grade point average. P-values were calculated using T-test. *Significant p-value (<0.05)

Hig-sugar beverages			
Times per week/GPA	2.3-2.99	3.0-3.59	3.6-4.0
<1	5 (24 %)	25 (36 %)	21 (42%)
1/wk	4 (19 %)	7 (10 %)	6 (12 %)
2-3/wk	3 (14 %)	19 (27 %)	10 (20 %)
4-6/wk	1 (5 %)	8 (11 %)	6 (12 %)
1/day	7 (33 %)	6 (9 %)	4 (8 %)
2+/day	1 (5 %)	5 (7 %)	3 (6 %)
Fast Food			
Times per week/GPA	2.3-2.99	3.0-3.59	3.6-4.0
<1	9 (43 %)	44 (63 %)	29 (58 %)
1/wk	7 (33 %)	9 (13 %)	14 (28 %)
2-3/wk	4 (19 %)	10 (14 %)	4 (8 %)
4-6/wk	0 (0 %)	3 (4 %)	1 (2 %)
1/day	1 (5 %)	3 (4 %)	2 (4 %)
2+/day	0 (0 %)	1 (1 %)	0 (0 %)
Fruit (%)			
Times per week/GPA	2.3-2.99	3.0-3.59	3.6-4.0
<1	2 (10 %)	8 (11 %)	5 (10 %)
1/wk	0 (0 %)	13 (19 %)	3 (6 %)
2-3/wk	11 (52 %)	14 (20 %)	13 (26 %)
4-6/wk	4 (19 %)	16 (23 %)	10 (20 %)
1/day	2 (10 %)	6 (9 %)	6 (12 %)
2+/day	2 (10 %)	13 (19 %)	13 (26 %)
Leafy greens and vegetables (%)			
Times per week/GPA	2.3-2.99	3.0-3.59	3.6-4.0
<1	1 (5 %)	4 (6 %)	0 (0 %)
1/wk	0 (0 %)	4 (6 %)	4 (8 %)
2-3/wk	6 (29 %)	20 (29 %)	13 (26 %)
4-6/wk	7 (33 %)	16 (23 %)	13 (26 %)
1/day	2 (10 %)	12 (17 %)	10 (20 %)
2+/day	5 (24 %)	14 (20 %)	10 (20 %)
Diet p-values	*0.1621	**0.1084	***0.0425

When assessed via academic achievement, 33% of LAA students consumed high-sugar beverages once a day, a stark contrast to the 36% of MAA and 42% of HAA students that consumed high-sugar beverages less than once a week. Following a similar trend, fast-food consumption at least once a week was greater in the LAA students (33%) than those in the HAA students (28%). Fast-food consumption of less than once per week was greatest in the MAA students (63%) and HAA students (58%).

Assessing the effect of healthy diets based on fruit consumption demonstrates that 19% of LAA students reported eating fruit more than four times a week versus 23% and 20% of MAA and HAA students, respectively. The data showed leafy greens and vegetables were consumed slightly more by HAA students versus LAA students. About 40% of HAA students reported eating leafy greens and vegetables at least once per day compared to the 34% of LAA students who reported eating leafy greens and vegetables at least once per day. Overall, the data showed a statistically significant positive correlation between diet and academic performance when comparing the HAA and LAA students (p = 0.0425).

Physical activity

The assessment of physical activity among first- and second-year medical students was focused on the intensity and frequency of exercise based on the SLIQ questionnaire. The focus in intensity was divided into light (e.g., leisurely walking, insubstantial housework), moderate (e.g., bicycling, swimming, dancing), and heavy exercise (e.g., weight training, soccer, basketball, aerobic workouts). The percentages of each of the exercise groups were then based on their academic standing. When analyzing the exercise intensity and frequency between the LAA group and the HAA groups, the data revealed a positive correlation. Overall, as exercise intensity increased, the number of HAA participants also increased (p = 0.0117).

Among the light exercise group, most students in the low achievement academic group (52%) reported participating in light exercise 1-3 times a week. In comparison, those with high academic achievement (40%) were participating in light exercise 4-7 times a week.

In the moderate exercise group, most students participated in physical activity 1-3 times a week (62%, 53%, and 46%) despite their level of academic achievement.

The last category of exercise included high-intensity activities. Based on the results, 40% of HAA students engaged in more intense activities 4-7 days a week in comparison to the 24% and 27% of LAA and MAA students, further exemplifying that higher exercise intensity correlated with higher academic achievement (Table 3).

**Table 3:** The Association Between Physical Activity and the Study Variables <1: less than once a week; 1-3/wk: 1-3 times per week; 4-7/wk: 4-7 times per week; 1+/day: 1 or more times per day; GPA: grade point average. P-values were calculated using T-test. *Significant p-value (<0.05)

Light Exercise (%)			
Times per week/GPA	2.3-2.99	3.0-3.59	3.6-4.0
<1 wk	2 (10 %)	6 (9 %)	3 (6 %)
1-3/wk	11 (52 %)	28 (40 %)	20 (40 %)
4-7/wk	6 (29 %)	20 (29 %)	20 (40 %)
1+/day	2 (10 %)	16 (23 %)	7 (14 %)
Moderate exercise (%)			
Times per week/GPA	2.3-2.99	3.0-3.59	3.6-4.0
<1 wk	5 (24 %)	15 (21 %)	9 (18 %)
1-3/wk	13 (62 %)	37 (53 %)	23 (46 %)
4-7/wk	3 (14 %)	16 (23 %)	15 (30 %)
1+/day	0 (0 %)	2 (3 %)	3 (6 %)
Heavy exercise (%)			
Times per week/GPA	2.3-2.99	3.0-3.59	3.6-4.0
<1 wk	6 (29 %)	17 (24 %)	12 (24 %)
1-3/wk	10 (48 %)	33 (47 %)	18 (36 %)
4-7/wk	5 (24 %)	19 (27 %)	20 (40 %)
1+/day	0 (0 %)	1 (1 %)	0 (0 %)
Exercise p-values	*0.0767	**0.2311	***.0117

Lifestyle satisfaction

Stress levels of participants since starting medical school were determined via a 1-9 point scale, 9 being the most stressed and 1 being the least. The average stress of LAA students was found to be 7.23 versus the HAA students with 6.16 stress levels, indicating a negative correlation between stress levels and academic achievement. Interestingly, 38% of LAA students agreed that they do sacrifice study time to exercise in contrast to the 42% of HAA students. This data supports that sacrificing study time to exercise may have a positive impact on academic performance.

To supplement these findings, satisfaction with mental health, physical health, and academic achievement was also assessed in the survey. About 19% of LAA students strongly disagreed that they were satisfied with their mental health, while 0% strongly agreed that they were satisfied. Remarkably, 52% LAA students disagreed that they were satisfied with their mental health. HAA students were more likely to strongly agree (16%) and agree (58%) that they were satisfied with their mental health.

A similar dissatisfaction among LAA students was also found when it came to reporting satisfaction with their physical health and academic achievement. About 24% and 48% of LAA students strongly disagreed or disagreed that they were satisfied with their physical health. Further, 43% of LAA students disagreed that they were satisfied with their academic performance. In contrast, 42% of HAA students agreed that they were satisfied with their physical health, and 60% agreed that they were satisfied with their academic achievement (Table 4).

**Table 4:** The Association Between Lifestyle Satisfaction Indicators and Study Variables SD: Strongly disagree; D: disagree; A: agree; SA: strongly agree. P-values were calculated using T-test. *Significant p-value (<0.05)

Question	Response	2.30-2.99	3.00-3.59	3.60-4.00
Overall stress level	Rating 1-9	7.23	6.55	6.16
Sacrifice study time to exercise	SD	3 (14 %)	14 (20%)	6 (12%)
	D	8 (38 %)	21 (30%)	15 (30 %)
	A	8 (38 %)	20 (29 %)	21 (42 %)
	SA	2 (10 %)	15 (21 %)	8 (16 %)
Satisfied with mental health	SD	4 (19 %)	13 (19 %)	12 (24 %)
	D	11 (52 %)	12 (17 %)	1 (2 %)
	A	6 (29 %)	33 (47 %)	29 (58 %)
	SA	0 (0 %)	12 (17 %)	8 (16 %)
Satisfied with physical health	SD	5 (24 %)	15 (21 %)	2 (4 %)
	D	10 (48 %)	22 (31 %)	21 (42 %)
	A	6 (29 %)	26 (37 %)	21 (42 %)
	SA	0 (0 %)	12 (10 %)	6 (12 %)
Satisfied with academic achievement	SD	5 (24 %)	3 (4 %)	0 (0 %)
	D	9 (43 %)	18 (26 %)	1 (2 %)
	A	7 (33 %)	40 (57 %)	30 (60 %)
	SA	0 (0 %)	9 (13 %)	19 (38 %)
Lifestyle p-values		0.0152*	0.0987**	0.0007***

## Discussion

Current literature has identified several key lifestyle factors that are characteristic of a healthy individual. These include increased consumption of fruit/vegetables and home-cooked meals, decreased consumption of high-sugar beverages and fast food, as well as the frequency of participation in physical activity. These elements can typically give a glimpse into the overall healthfulness of one’s life, and thus, they were assessed in this study. The results of this study are in alignment with current literature and are supported by the previously presented data showcasing an increase in healthy lifestyle habits in HAA students compared to LAA students.

Nutrition dietary factors

The importance of maintaining a well-balanced diet rich in all vegetables, fruits, lean proteins, and healthy fats is often a topic discussed during many nutrition courses in medical school. Interestingly though, Likus et al. found in a study examining nutritional behavior among medical professional students that only 29% of students had daily fruit and vegetable consumption, while 45.6% reported snacking on potato chips and salty foods between meals [3]. Although the results were not compared to the students’ academic performance, it illustrates that medical students are not always putting into practice what they learn. In the current study, it was found that the HAA students (3.60-4.00 GPA) consumed more fruit, vegetables, and home-cooked meals compared to the middle (3.00-3.59 GPA) and low (2.30-2.99 GPA) AA students, while the LAA students consumed high-sugar beverages at the highest rate and HAA students at the lowest. Responses for healthy dietary habits were statistically significant across all categories, where HAA students participated in healthy habits the most and LAA participated the least with moderate MAA students in the middle.

The data show that healthier dietary habits may be crucial for brain functioning and consequent academic achievement. A diet filled with low-quality fats, sugars, and highly processed macronutrients can lead to decreased energy, dysregulated appetite/mood, and an overall reduction in mental clarity. Long-chain saturated fatty acids pass directly into the hypothalamus producing an inflammatory response and have been shown to impair hippocampus-dependent memory function in humans and rodents [7]. One of the simplest ways in which medical students negatively impact their nutrition is through the consumption of beverages with high-sugar content, such as energy drinks, soft drinks, and coffee with added sugar. A Polish study found that energy drinks were consumed by 39% of medical students daily, and 40% reported daily consumption of sweetened beverages [3]. In comparison, the International Journal of Health Sciences found that 77.4% of students consumed soft drinks at least three times per week with 94.1% understanding the disadvantages [8]. Although sweetened beverages and energy drinks may help medical students stay awake longer or provide a boost in energy, they come with a host of negative effects. Studies have shown that energy drinks are linked to anxiety and depression, sleep disturbances, neurological and cardiovascular damage, and substance abuse [9]. This notion is supported by the findings in this study showing that the HAA students did not rely on high-sugar beverages as much as LAA or MAA students.

Home-cooked meals offer more control of caloric intake, fat, sugar, and added preservatives to meals than those purchased from restaurants or fast-food chains, while typically having more whole, nutrient-dense ingredients. This allows consumers to reduce unfavorable habits like overconsuming fast food, unhealthy fats, and calories. Research has shown that colorful plants such as fruits or vegetables contain polyphenolic compounds which have potent antioxidant and anti-inflammatory activities, including decreased neuroinflammation [6]. In contrast, foods that contain high concentrations of saturated fats and simple carbohydrates, for instance, those found in fast food and ready-made meals, are associated with increased oxidative stress and lower the levels of neurotrophic substances derived from the hippocampus [10]. Even though healthy options exist at fast-food and fast-casual chains, previous studies have illustrated that students are not choosing these options. In 2021, the sustainability survey of 403 medical students found that 82.38% consumed fast food with their favorite options including pizza, hamburgers, fries, and breaded chicken strips [11]. The trend in our results of increasing consumption of home-cooked meals as AA increases strongly supports our hypothesis that HAA students eat more balanced and nutritious meals allowing them to be better fueled for studying and test taking while evading the detrimental side effects of diets with low-quality ingredients, including high glycemic indices, postprandial hypoglycemia, high cholesterol, and saturated fats.

Physical activity

Based on our questionnaire results, physical activity at all intensity levels had a statistically significant positive correlation with AA (p = 0.012), therefore demonstrating that increased physical activity among medical students in this sample correlates with obtaining higher AA. Our results found that medical students who reported higher physical activity habits also had a higher GPA (p = 0.001). A similar correlation was found between physical activity and a higher GPA in the fourth- and fifth-year students in a cross-sectional study conducted in Saudi Arabia [12]. In further agreement with our results, it has been found that 2-3 hours of physical activity per week is positively correlated with academic success [13].

Apart from the physical benefits of working out, physical activity increases cognition by stimulating an increase in blood flow to the brain. Cerebral blood flow is increased with an increase in cardiac output, which facilitates cerebral perfusion; therefore, higher-intensity workouts have been associated with increased short-term memory [2]. Aerobic exercise can enhance memory due to the increase in insulin-like growth factor-1 (IGF-1), which influences the rate of neurogenesis and formation of new neurons. Additionally, another mechanism by which physical activity can impact memory is via the increase in brain-derived neurotrophic factor (BDNF), which stimulates neuroplasticity throughout the cortex, but most specifically the hippocampus [14]. Changes in neuroinflammatory cytokines have also been measured with an overall reduction in pro-inflammatory cytokines such as IL-6 and an increase in anti-inflammatory cytokines promoting cognitive function and memory. Further, alterations in synaptic plasticity and spine density can also play a positive role in mediating the effects of physical activity on learning and memory [15].

The data show that 40% of students in the HAA group performed heavy exercise at least four times a week, compared to only 24% of LAA medical students. Although the difference between academic performance and physical activity levels were not analyzed based on gender, former studies have shown a significant difference for males vs females. For instance, Chung et al. found that males were involved in health-enhancing physical activity more than females, and the odds of having a good GPA was twice as high among those involved in physical activity [16]. Overall, the results of the study show that an increase in physical activity is positively correlated to higher academic achievement which is further supported by the physiological concept of increased cerebral blood flow and neurogenesis on cognition and short-term memory. 

Lifestyle satisfaction

An important aim of this study was to not only assess factors that contribute to academic achievement but to also explore how students perceive their satisfaction with their own mental health, physical health, and academic achievement. A total of 0% of LAA students responded “strongly agree” to the three questions asking about satisfaction with mental health, physical health, or academic achievement. This highlights an unfortunate correlation that students with the unhealthiest lifestyle practices have the lowest GPAs and lowest satisfaction with their mental and physical health. Decreased life satisfaction can cause various levels of psychological distress especially when associated with circumstances outside of one’s control such as the COVID-19 pandemic. For instance, students in Lebanon during COVID-19 reported greater anxiety, insomnia, and depression, all of which can negatively impact academic performance despite healthy behaviors [17]. In comparison, 38% and 12% of HAA students responded “strongly agree” to satisfaction with academic achievement and physical health, respectively. About 71% of the LAA cohort expressed dissatisfaction with their mental health, 72% expressed dissatisfaction with their physical health, and 67% expressed dissatisfaction with their academic achievement, while 98% of HAA students expressed satisfaction with their academic achievement, 54% expressed satisfaction with their physical health, and 74% expressed satisfaction with their mental health. Although it was not assessed in this study, previous research has found that students that reported more psychological distress and increased frequency of eating out was significantly associated with lower subjective academic achievement scores; illustrating the interplay between diet, lifestyle satisfaction, and academic achievement [18].

It is evident that there is a disparity in lifestyle satisfaction among the AA cohorts in this study. Although the researchers acknowledge that this could be due to several factors, one thing remains clear, those with healthier lifestyles including physical activity and nutrient-rich eating habits reported a higher satisfaction with their health and achievement. A healthier lifestyle and increase in lifestyle satisfaction can positively impact self-esteem as well. Those with a positive self-esteem are able to manage developmental responsibilities such as academics, social interactions, and personal needs, which allows individuals to develop increased career decision self-efficacy with decreased rates of psychological distress [19]. The idea of lifestyle satisfaction is not unfounded but is a means to analyze the ever-growing epidemic among medical students and healthcare professionals: burnout. The results of this study show that maintaining healthy habits can potentially be protective against burnout which may lead to more empathetic, engaged, and equipped healthcare professionals.

To our knowledge, this is the first study analyzing the impact of healthy lifestyle factors on academic achievement in medical school while also inquiring about overall satisfaction with health and academic achievement among medical students in the United States. The researcher's initial hypothesis believed that increasing studying hours at the expense of healthy lifestyle practices would not increase academic achievement due to the concept of diminishing returns. However, the data show there is not a major variation in hours spent studying between different levels of academic achievement, suggesting that variations in studying time do not play such an impactful role in academic achievement as we once thought. However, it is healthy lifestyle practices working in synergy that have the strongest correlation with increased academic achievement and lifestyle satisfaction regardless of time studying.

It is important to note that other factors may be at play regarding the correlation between healthy lifestyle practices and increased academic achievement. Those who find time to exercise and cook healthy meals despite the rigorous schedule of a medical student may possess certain qualities (time management, organization skills, highly efficient) that lend to their success in academics regardless of their participation in healthy lifestyle practices. The authors recognize this as a potential confounding variable in the study and believe that future research is needed to further delineate whether it is the direct effect of a healthy lifestyle vs the intrinsic characteristics of a health-conscious person that contributes to increased academic achievement, or a combination of both.

Study limitations

This study faced several limitations that should be noted. The participant pool assessed consisted primarily of first-year medical students (82%) compared to second-year medical student responses (18%). The responses recorded were also unbalanced in terms of gender, with more female responses (60%) to male responses (40%). This potentially skews the results toward the perspectives and experiences of female OMS-I students, further limiting the reliability and generalizability of the data collected. Moreover, the study was standardized to include only one DO medical school, thereby neglecting variations in curriculum seen at other medical schools. This narrow focus may limit the extrapolation of the findings to other institutions, as the unique elements of NSU-KPCOM’s curriculum and location could have a significant bearing on how diet, exercise, and lifestyle impact academic performance.

Future research

Future research is warranted to determine whether the findings of this study apply to schools observing a pass/fail curriculum, allopathic medical schools, and medical students beyond their didactic years. Expanding this study to include multiple medical schools throughout different geographical locations with varying student demographics would provide information on our findings' reproducibility. Future experimental studies could shed light on the best ways to implement the lifestyle habits that we found to be most conducive to success in medical school, specifically, how to implement more home cooking and physical activity into the busy schedules of medical students. Observational studies comparing the longevity, satisfaction, and clinical performance of high, medium, and low AA medical students and their self-reported lifestyles while in medical school and beyond would be an interesting longitudinal application of this study that would provide novel data on the career paths and patient care provided by each category of student.

## Conclusions

The rigor and competitiveness of medical school are unlikely to change, but the mindset and strategies to handle the lifestyle can. With a focus on increasing healthy food choices and consistent physical activity, medical students can be better prepared to handle the stresses that accompany a life in medicine. The data collected from this study can help better equip and educate medical students on the benefits of effective work-life balance. Healthy lifestyle practices are not only beneficial to one’s physical and mental health but may also play a key role in physiological processes that can improve cognitive functioning and consequently academic achievement. The impact of this study is not limited to raising awareness that medical students should adopt healthier habits, a concept most people understand. Moreover, it aimed to analyze the interplay of healthy lifestyle habits more deeply and how they produce a higher achieving and an emotionally satisfied student. The researchers are not merely concluding that adequate nutrition and physical activity should be increased among medical students, as much of the current literature has concluded already. Instead, they are proposing these intentional habits are essential to high-functioning medical students and possibly play a key role in their longevity and achievement as students and practicing physicians.
